# The relationship between self-efficacy, resilience, and job burnout in pediatric residents: a cross-sectional study in Western China

**DOI:** 10.1186/s12909-024-05700-y

**Published:** 2024-07-23

**Authors:** Yuxi Du, Lina Qiao, Liqun Dong, Chaomin Wan, Xue Yang, Hanmin Liu

**Affiliations:** 1 Key Laboratory of Birth Defects and Related Diseases of Women and Children (Sichuan University), Ministry of Education, Chengdu, China; 2 Sichuan Children’s Clinical Research Center, Chengdu, China; 3 President’s Office, West China Second University Hospital, Sichuan University, Jinjiang District, Chengdu, Sichuan province China; 4 Pediatric Department, West China Second University Hospital, Sichuan University, Chengdu, China

**Keywords:** Resilience, Job burnout, Self-efficacy, Pediatric resident, Western China

## Abstract

**Background:**

Burnout is prevalent among pediatric residents. Self-efficacy and resilience, as concepts of positive psychology, may be protective factors for burnout. However, no current data demonstrates the mechanism of their interaction.

**Objectives:**

To investigate the pediatric residents’ status of self-efficacy, resilience, and job burnout in a university-affiliated hospital in western China. To explore relationships among them, especially the mediating effects of resilience.

**Methods:**

The study was conducted with 190 pediatric residents from an A-Class women’s and children’s hospital in western China. Data included demographic characteristics, status of pediatric residents, measures of burnout (using the Physicians’ Career Burnout Questionnaire), self-efficacy (using the General Self-Efficacy Scale) and resilience (using the Connor-Davidson Resilience Scale). Multiple regression analysis and mediation analysis with bootstrapping were used to identify whether resilience mediates the relationship between self-efficacy and burnout.

**Results:**

Female pediatric residents exhibited significantly lower self-efficacy (t = 2.53, *p*<0.05) and higher levels of job burnout (t=-2.64, *p*<0.01) compared to male residents. Residents in the standardized training stage experienced higher levels of job burnout compared to those who had completed the training, as indicated by t-values of -3.21, -2.13, and − 2.80 (*p*<0.05). Significant correlations (*p* ≤ 0.01) were found among self-efficacy, resilience, and burnout. Additionally, our findings indicated that pediatric residents’ self-efficacy can positively predict job burnout and its three dimensions through a major mediating effect of resilience.

**Conclusions:**

The findings regarding the mediating effect of resilience on the influence of self-efficacy on burnout, and their association with gender and residency status, have practical implications for interventions aimed at reducing burnout and improving the well-being of pediatric residents.

**Supplementary Information:**

The online version contains supplementary material available at 10.1186/s12909-024-05700-y.

## What’s new

We investigated pediatric residents’ self-efficacy, resilience, and burnout in China. Self-efficacy and mental resilience have a significant correlation with burnout, and resilience plays a mediating role in this relationship. Understanding the interaction helps us identify interventions to improve residents’ mental health.

Job burnout among healthcare professionals is defined as a syndrome of “various degrees of emotional exhaustion, depersonalization and a low sense of personal accomplishment [1]”. Burnout is prevalent during medical education, with major US multi-institutional studies estimating that at least half of medical students [2] and resident trainees [3] are affected. Similarly, pediatric residents in China face significant challenges in this regard [4]. Firstly, job burnout leads to physical and mental exhaustion, which reduces the quality of life and even produces the desire or action of quitting and even suicide [5]. Secondly, job burnout not only reduces work efficiency but also negatively impacts medical service quality. This can affect the doctor-patient relationship and even threaten the health of child patients [6]. Compared with adult patients, diagnosing pediatric diseases accurately and timely poses greater challenges due to the rapid progression and changes in pediatric conditions [7]. Pediatricians also face lower income, high-intensity work, conflicts with patients, and a multitude of clinical and theoretical tests, which contribute to significant occupational pressures and hence more severe burnout [8].

Resilience is defined as the ability of individuals to bounce back or to cope successfully with adverse circumstances [9]. Studies have shown resilience is one of the protective factors of job burnout [10]. While studies have explored resilience among healthcare workers [11], family physicians [12], and pediatric intensive care unit staff [13], highlighting the need for improvement, only a few of studies have focused on the resilience of pediatric residents. These studies explored the relationship between self-compassion, mindfulness [14], well-being [15] and the resilience of pediatricians, without mentioning job burnout. As a critical and important stage in a pediatrician’s career, residency represents a controllable educational period for interventions aimed at cultivating the resilience of future pediatricians as a long-lasting beneficial personal quality. Thus, investigating the current situation and relationship between resilience and job burnout among pediatric residents is important, providing evidence for enhancing their mental health.

Self-efficacy is an individual’s belief in their ability to plan and execute a course of action, which is hypothesized to influence behaviors and environments, and in turn, to be influenced by them [16]. In the medical field, while literature regarding self-efficacy among pediatric residents predominantly examines their medical skills and behaviors, such as resuscitation [17] and medical knowledge [18], only a few studies have explored generalized self-efficacy as an intermediary variable in the context of subjective well-being [19] and occupational stress. The corresponding impact of self-efficacy in pediatric residents is not clear and further insight is necessary to address current adversities.

In pediatric residents, no literature reveals the dynamic correlation among burnout, resilience, and self-efficacy. Based on the previously mentioned research foundation, we hypothesize that resilience mediates the relationship between self-efficacy and job burnout. To fill the research gaps, this study aimed to conduct a comprehensive cross-sectional analysis to investigate the status of self-efficacy, resilience, and job burnout in pediatric residents with a sample from a Class A children’s hospital in western China and to characterize the correlations and possible mechanisms among these variables.

## Methods

### Participants and procedures

To address our research questions, we conducted an online survey with pediatric residents from a highly regarded Class A women’s and children’s hospital in western China. Affiliated with a prestigious university, this hospital consistently ranks among the top ten in Fudan University’s children’s hospital specialty ranking. It emphasizes medical treatment, teaching, and scientific research, with a particular focus on academic achievements. The questionnaire link was distributed to all pediatric residents, including those who had graduated with at least a bachelor’s degree from their 3-year standardized medical training at the hospital, as well as those currently undergoing the training. We requested their voluntary participation in the study. The informed consent form assured the pediatric residents that there would be no conflicts of interest or disadvantages associated with their involvement and emphasized their right to participate voluntarily. A total of 200 pediatric residents were recruited for this cross-sectional survey in November 2022.and 190 valid questionnaires were collected, resulting in a response rate of 95%. Table 1 provides an overview of the participants’ gender, education level, standardized training status, and occupation title.

**Table 1:** Characteristics of participants

Variable	Sub-Variable	Number of Cases(*N*)	Percentage (%)
Gender	male	31	16.2
	female	159	83.8
Education level	bachelor	65	34
	master	101	52.9
	PhD	24	13.1
Grade (standardized training)	finished	90	47.4
	1st year	41	21.5
	2nd year	27	14.2
	3rd year	32	16.8
	Total	190	100

### Instruments

#### Job burnout - physicians’ career burnout questionnaire (PCBQ-PMI)

In this study, the Physicians’ Career Burnout Questionnaire (PCBQ-PMI) compiled by Zhang Yimin [20], was used to assess the job burnout of the subjects. This scale is based on the Chinese Maslach Burnout Inventory (CMBI,21 items) compiled by Li Yongxin et al. [21]. The PCBQ-PMI questionnaire consists of 11 items, categorized into three dimensions: emotional exhaustion (5 items), depersonalization (3 items) and low career accomplishment (3 items). Respondents rate each item on a 5-point scale, ranging from 1 (‘not true at all’) to 5 (‘true nearly all the time’). Higher scores indicate a higher degree of burnout experienced by the participants in their current job. The PCBQ-PMI demonstrates good internal consistency with a Cronbach’s alpha of 0.848.

#### Resilience - connor-davidson resilience scale(CD-RISC, 10-items version)

The Connor-Davidson Resilience Scale (CD-RISC) is a well-used instrument for measuring resilience [22]. Campbell-Sills and Stein made a series of empirical modifications to the CD-RISC, resulting in a 10-item unidimensional scale [23]. The revised 10-item CD-RISC has good internal consistency (Cronbach’s alpha = 0.85). Each item is rated on a 5-point scale from 1 (‘not true at all’) to 5 (‘true nearly all the time’).

#### Self-efficacy - general self-efficacy scale (GSES)

General Self-Efficacy Scale (GSES) is primarily used to measure the broadest range of self-efficacy in a non-specific field. The scale was proposed in 1982 by the German psychologist Schwarzer [24] and was later translated and revised by Caikang Wang et al. [25] in Chinese populations. This Chinese version contains 10 items and uses a 4-point scoring system. Each item is rated on a 4-point scale from 1 (‘not true at all’) to 4 (‘true nearly all the time’). The GSES is a unidimensional scale that calculates a total score, where a higher total score indicates stronger general self-efficacy. The correlation coefficient between the 10 items of GSES and the total quantity table ranges from 0.60 to 0.77, demonstrating good reliability and validity in the Chinese context. In this study, the internal consistency coefficient of the scale is reported as 0.83, indicating good reliability.

### Data analysis

We used SPSS Statistics 24 software for basic analysis of the collected responses by conducting descriptive statistical analysis (mean and standard deviation) and correlation analysis. Additionally, to explore the relationship among the three factors mentioned, we formulated a theoretical causal mediation hypothesis. Instead of conducting a simple binary correlation analysis between every two factors, mediation analysis incorporating all three factors may reveal the process and mechanism of how one factor is related to another. We conducted a mediating effect analysis using the PROCESS plug-in, according to Hayes Model 4 [26] (a simple intermediary model). The mediating effect of resilience in the relationship between self-efficacy and job burnout (including emotional exhaustion, depersonalization and low career accomplishment) was tested while controlling for identity, gender, education background, and title. Specifically, ordinary least squares regressions were used to test the effect with a stepwise approach [27], namely the test of total effect, test of joint significance [28], and the test of direct effect. In the key step of test of joint significance, Bootstrap method [29] was used to obtain a 95% confidence interval.

## Results

Descriptive and Correlation Analysis among Pediatric Residents’ Emotional Exhaustion, Depersonalization, Low Career Accomplishment, Resilience, and Self-efficacy.

Descriptive statistical analysis is shown in Table 2. For job burnout(PCBQ-PM), it was demonstrated that the mean of emotional exhaustion(3.40 ± 0.83) is statistically higher than depersonalization(2.89 ± 0.86) (t = 10.30, *P* < 0.05 paired T test) and low accomplishment(2.63 ± 0.93) (t = 13.61, *P* < 0.05 paired T test). A correlation analysis was performed to determine if the five variables were interrelated. The results revealed statistically significant correlations among all five variables (*p* ≤ 0.01) (Table 2).

**Table 2:** The correlation between burn-out, resilience and self-efficacy among pediatric residents

Scales	Subscales	Mean ± D (*n* = 190)	Burnout-Emotional exhaustion	Burnout-Depersonalization	Burnout-Low career accomplishment	Resilience	Self-efficacy
PCBQ-PMI (5-point)	Burnout-emotional exhaustion	3.40 ± 0.83	1	0.670^**^	0.618^**^	-0.324^**^	-0.251^**^
	Burnout-depersonalization	2.89 ± 0.86	0.670^**^	1	0.679^**^	-0.435^**^	-0.349^**^
	Burnout-Low career accomplishment	2.63 ± 0.93	0.618^**^	0.679^**^	1	-0.515^**^	-0.394^**^
GSES (5-point)	resilience	3.45 ± 0.68	-0.324^**^	-0.435^**^	-0.515^**^	1	0.718^**^
CD-RISC (4-point)	Self-efficacy	2.44 ± 0.58	-0.251^**^	-0.349^**^	-0.394^**^	0.718^**^	1

*p<0.05, **p<0.01

Table 3 shows that in terms of gender, the efficacy of female pediatric residents is significantly lower than that of male residents(t = 2.53,*p*<0.05), while the job burnout is significantly higher than that of male counterparts(t=-2.64, *p*<0.01). There was no significant difference in the three aspects (emotional exhaustion, depersonalization, and low career accomplishment) among the people with different education levels(*p*>0.05). In terms of standardized training, the job burnout of residents who completed standardized training was significantly lower than that of pediatric residents who had not completed standardized training(post hoc comparisons with each grade shows t=-3.21, -2.13, -2.80, all with *p*<0.05), and resilience of pediatric residents who completed routine training was significantly higher than that of residents in the first year of standardized training(t=2.86, *p*<0.01).

**Table 3:** Average level of self-efficacy, resilience, and Job burn-out by demographic variable

Variable	Sub-Variable	Self-efficacy	Resilience	Burnout-All
Gender	male	2.68 ± 0.63	3.66 ± 0.83	2.72 ± 0.91
	female	2.40 ± 0.56	3.41 ± 0.64	3.11 ± 0.71
	T test	2.53*(0.012)	1.94(0.054)	-2.64**(0.009)
Education level	bachelor	2.42 ± 0.57	3.43 ± 0.78	3.14 ± 0.81
	master	2.47 ± 0.61	3.47 ± 0.65	2.95 ± 0.74
	PhD	2.40 ± 0.47	3.42 ± 0.50	3.23 ± 0.66
	ANOVA & LSD	0.21(0.814)	0.10(0.906)	1.98(0.141)
grade (standardized traning)	Finished	2.53 ± 0.49	3.56 ± 0.57	2.83 ± 0.62
	1st year	2.30 ± 0.75	3.20 ± 0.86	3.28 ± 0.81
	2nd year	2.42 ± 0.63	3.48 ± 0.75	3.18 ± 1.00
	3rd year	2.43 ± 0.49	3.44 ± 0.60	3.28 ± 0.68
	ANOVA & LSD	1.42(0.239)	2.75*(0.044) (Finished > 1 year t = 2.86*)	5.04**(0.002) (Finished < 1,2,3 year t=-3.21*,-2.13*,-2.8*)

Value in t test means t value in independent samples test; Value in ANOVA means F value. If F value is significant then LSD (Least Significant Difference) post hoc test is conducted to compare the difference between any two groups. The group pairs with significant differences and corresponding t value are showed after F value. P values are added to the t or F values in brackets

** *p* < 0.01 * *p* < 0.05

### The full mediating effect of resilience between job burnout and self-efficacy

**Fig. 1:**
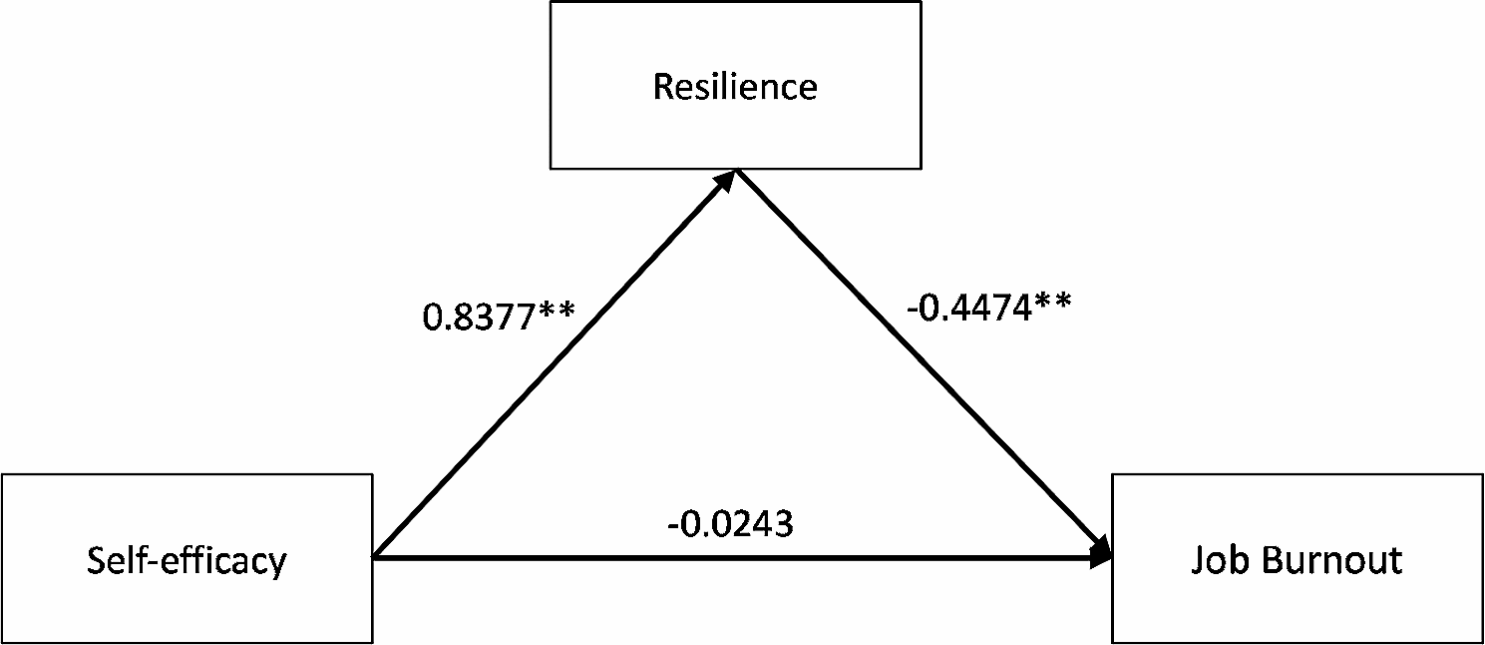
Resilience mediating effect model of job burnout *p<0.05, **p<0.01

**Fig. 2:**
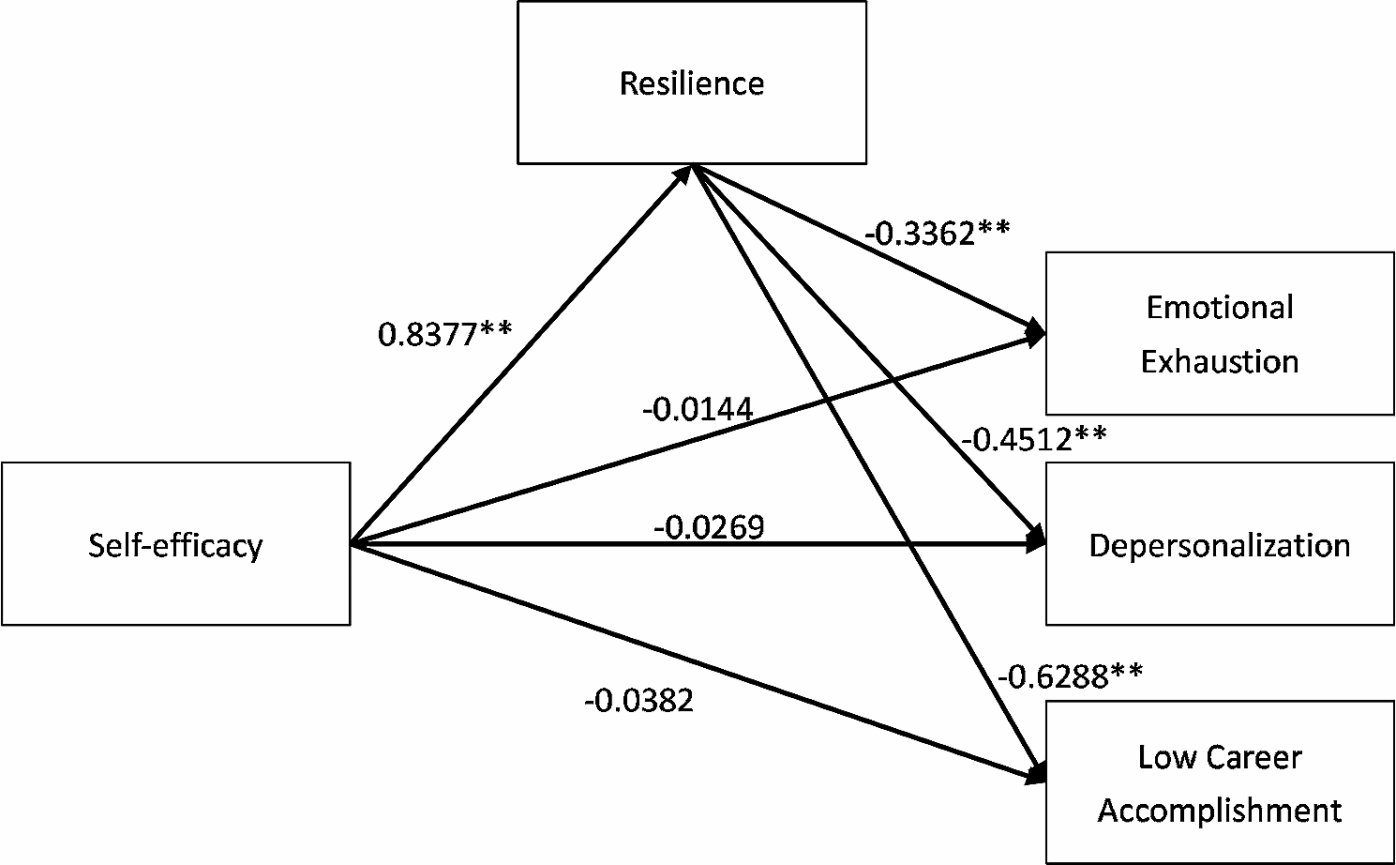
Resilience mediating effect model of the three dimensions(emotional exhaustion, depersonalization, accomplishment) of job burnout *p<0.05, **p<0.01

Results (Table 4) indicated that the self-efficacy of residents had a significant correlation relationship on job burnout and its three dimensions (β = 0.399 (0.296, 0.405, 0.565), t = 4.499**, (2.874 **, 4.077 **, 5.190 **)), *p* < 0.01). However, when the intermediate variable resilience was added, the direct effect of self-efficacy on job burnout was not significant (β =-0.024(-0.014,-0.027,-0.038).t=-0.204(-0.101,-0.200,-0.266), *p* > 0.05). Self-efficacy was significantly positively related to resilience (β = 0.838, t = 13.439**, *p* < 0.01). The negative effect of resilience on job burnout and its three dimensions was also significant (β = 0.447 (0.336, 0.451, 0.629), t = 4.481 ** (2.811 **, 3.996 **, 5.222 **), *p* < 0. 01). Additionally, Table 5 shows that the upper and lower limits of the bootstrap 95% confidence interval for the direct effect of efficacy on job burnout and its three dimensions include 0, while for the mediating effect of the resilience does not contain 0, indicating that efficacy is not directly related to job burnout. However, it can predict job burnout and its three dimensions through the complete mediating effect of resilience. The mediating effect (-0.375, (-0.282, -0.378, -0.527)) accounted for 94%, (95%, 93%, 93%) of the total effect (-0.399, (-0.296, -0.405, -0.565)). The mediating models with job burnout and its three dimensions are summarized in Figs. 1 and 2.

**Table 4:** The mediating effect of resilience

Resulting Var.	Predictive Var.	*R*	*R*^2	F	Beta	t	*p*
Job burnout	identity	0.475	0.226	10.725**	0.144	3.712**	0.0003
	gender				0.213	1.539	0.1255
	education				0.135	1.514	0.1322
	title				-0.012	-0.239	0.8114
	self-efficacy				-0.399	-4.499**	0.0000
Resilience	identity	0.720	0.518	39.561**	-0.026	-0.959	0.3388
	gender				-0.004	-0.036	0.9713
	education				-0.009	-0.150	0.8809
	title				0.019	0.546	0.5857
	self-efficacy				0.838	13.439**	0.0000
Job burnout	identity	0.550	0.302	13.211**	0.133	3.574**	0.0005
	gender				0.212	1.605	0.1102
	education				0.131	1.541	0.1250
	title				-0.003	-0.070	0.9443
	resilience				-0.447	-4.481**	0.0000
	self-efficacy				-0.024	-0.204	0.8386

**Table 5:** Testing the mediation model of resilience

Dependent variable		Effect	BootSE	BootLLCI	BootULCI	Effect Ratio
Job burnout	Total	-0.399	0.089	-0.574	-0.224	
	Direct	-0.024	0.119	-0.259	0.210	6%
	Indirect	-0.375	0.126	-0.614	-0.131	94%
Emotion exhaustion	Total	-0.296	0.103	-0.499	-0.093	
	Direct	-0.014	0.142	-0.295	0.267	5%
	Indirect	-0.282	0.134	-0.548	-0.011	95%
dispersonalization	Total	-0.405	0.099	-0.601	-0.209	
	Direct	-0.027	0.134	-0.292	0.238	7%
	Indirect	-0.378	0.135	-0.643	-0.118	93%
Low job accomplishment	Total	-0.565	0.109	-0.780	-0.350	
	Direct	-0.038	0.143	-0.321	0.245	7%
	Indirect	-0.527	0.138	-0.805	-0.265	93%

## Discussion

In our results, we found significant differences with demographic variables, significant correlations among self-efficacy, resilience, and burnout, and a mediation mechanism whereby pediatric residents’ self-efficacy can positively predict job burnout through a major mediating effect of resilience. Here, we first discuss the association between self-efficacy, burnout, and the key mediation effect, then briefly touch on grouped comparison results.

### The effect of self-efficacy on Pediatric residents’ job burnout

The results indicate a significant positive correlation between self-efficacy and job burnout, aligning with previous research findings across various occupations [30]. In our mediation hypothesis, we do not define self-efficacy as a confounder because self-efficacy beliefs have a prospective and operative character, and existing intervention studies have shown that self-efficacy-enhancing interventions would reduce employees’ strain [31]. There are two main aspects that explain the influence of self-efficacy on job burnout: firstly, self-efficacy reflects an individual’s subjective confidence [32] in their abilities. When pediatric residents feel more capable and confident in their skills, they tend to experience higher job satisfaction and a greater sense of achievement, leading to lower levels of burnout. Second, self-efficacy objectively represents the cognition of one’s own professional abilities [16]. If pediatric residents possess solid professional abilities, including research achievements, and are proficient in their work, they are more likely to handle tasks efficiently and effectively. This competence enables them to solve problems with ease and reduces the likelihood of experiencing negative emotions and burnout. Therefore, having a high level of self-efficacy can contribute to lower levels of job burnout among pediatric residents. Furthermore, it’s worth noting that this study was conducted in a children’s hospital affiliated with a renowned university. Such hospitals often have higher expectations for staff members regarding scientific research [33], which can introduce additional pressure. Based on this, efforts should be made to improve the scientific research environment in hospitals. This can be achieved through activities such as regular literature reading and sharing meetings to establish a scientific research platform with increased funding support for young residents.

### The full mediating role of pediatric residents’ resilience

Resilience emphasizes the capacity to bounce back from adversity and provides protection against workplace challenges, personal stressors, and the risk of burnout [34]. Regarding the direction, resilience is more stable and long-term than job-burnout so we assume that the primary direction of the relationship is that resilience affects burn-out, and not in reverse. Our results show that self-efficacy influences burnout predominantly through the mediating effect of resilience. The resulting mediating effect suggests that pediatric residents with high self-efficacy are more likely to be equipped with cognitive tools such as internal adjustment of resilience, actively facing and overcoming problems in professional life. This finding adds important supplementary and empirical evidence to the analysis of the mechanism of self-efficacy. Similar conclusions have been found in studies on faculty members in social and legal sciences [35] and students’ test anxiety [36] where resilience was also reported to mediate the effect of self-efficacy. Specifically, self-efficacy has a significant positive correlation effect on resilience. This can be attributed to the emotional aspect of self-efficacy directly affecting resilience. Residents with high efficacy tend to have more positive and optimistic emotions, which gives them more positive hints and confidence when facing difficulties. As a result, they exhibit stronger adaptability and resilience, aiding them in problem-solving and practice. This finding aligns with previous studies that identify self-efficacy as a protective factor for resilience.

On the other hand, the influence of resilience on job burnout can be understood in two aspects: first, the pediatric residents with strong resilience have a higher threshold for feeling burnout due to their strong tolerance and more positive and optimistic qualities; second, the mentally strong pediatric residents can quickly adapt and recover when encountering difficulties, making them less prone to burnout. The direct impact of resilience on coping with job burnout has been widely demonstrated in healthcare [10, 37]. Based on the above, self-efficacy strengthens its influence on job burnout through the major mediating effect of resilience. The research results suggest that in the education and training of pediatric residents, more attention should be paid to enhancing resilience, which can greatly reduce their job burnout and mediate the protective effects of self-efficacy.

### Analysis of differences in demographic variables

Besides the core mediation mechanism among self-efficacy, resilience, and job burnout, we also conducted grouped comparisons with demographic variables. The results showed that female pediatric residents reported lower efficacy and higher burnout compared to men. The reasons may include the unbalanced attention and opportunities of leadership for female physicians, more gender-specific work responsibilities, and the more challenging role of females in families and workplace, which may originate from the particularity of pediatrics, unbalanced gender distribution in pediatrics, and the patriarchal social structure at our observation [38, 39]. Policy support should be directed towards female residents, offering them increased opportunities for professional advancement and leadership positions [40].

The results also show that the professional efficacy of residents in the training stage is lower than that of those who have completed their training. Young pediatric residents often spend more time on tedious tasks such as writing medical records and handling administrative duties [41], which can lead to fatigue, a sense of weakness, and a low sense of achievement. Additionally, the lack of communication skills [42] and the limited depth and effectiveness of standardized training [43] further impact efficacy of young residents. Therefore, we recommend flexibly adjusting the training content and format to better cater to the needs of pediatric residents. The results reveal that first-grade residents have the lowest resilience. This can be attributed to their limited experience and lower efficiency in handling high-intensity work and emergency situations, indicating the necessity of early clinical practice and tutoring.

Medical education professionals are trying to find appropriate ways to effectively reduce burnout and improve students’ and residents’ self-efficacy and resilience. By integrating these topics into training curricula, residents can learn and practice positive resilience strategies that will serve them throughout their careers [44]. Besides, it is suggested that departmental humanistic activities^1^^,^ [45], such as critical event sharing, allow pediatric residents to learn from their own and others’ real experience and to have psychological expectations of various conditions in advance, improving their coping ability and resilience when they encounter difficulties. Mindfulness training^2^ [46] can also improve the department atmosphere, enhance team cohesion, cultivate strong personal mental abilities, and reduce job burnout. In some serious cases, pediatric residents should be provided with adequate psychological help and support through professional psychological counseling.

The study fills a gap in the existing literature by examining the relationship between job burnout, self-efficacy, and resilience among pediatric residents, shedding light on the significant correlation between self-efficacy and resilience in predicting job burnout. Additionally, the study reveals a major mediating role of resilience, providing insights into how self-efficacy influences job burnout through this pathway. The findings of the study hold practical significance for understanding job burnout among pediatricians, which can help in developing strategies to reduce job burnout and establish a healthier mental environment in the pediatric healthcare setting.

### Limitations

Our study solely relied on cross-sectional data, failing to capture the longitudinal dynamic changes in the resilience, self-efficacy, and job burnout of pediatric residents across different stages of their careers. Future research should consider longitudinal designs to provide a more comprehensive understanding of these variables over time. Additionally, the sample size and scope of participants were limited, with the survey being conducted in only one hospital in western China. To enhance the generalizability of the findings, subsequent studies are recommended to include a larger and more diverse sample.

## Conclusions

Residency is a formative experience, and residents often practice in the way they are trained [47]. It is critical to address concepts of wellness and burnout prevention during pre-employment training and standardized training periods. Therefore, conducting research on the factors influencing job burnout at an early stage is crucial for ensuring the long-term stability and well-being of pediatric residents. This study takes resilience and self-efficacy as the incision to provide a new perspective for pediatric burnout research. Our analysis of the potential mechanism has practical implications for interventions aimed at reducing burnout and improving the well-being of pediatric residents.

### Electronic supplementary material

Below is the link to the electronic supplementary material.

Supplementary Material 1

Supplementary Material 2

## Data Availability

All data generated or analyzed during this study are included in this published article and its supplementary information files.

## Footnotes

1 Wang Y, Zhang X, Xie Q, Zhou H, Cheng L. Humanistic caring ability of midwifery students in China and its associated factors: A multi-centre cross-sectional study. *Nurse Educ Today*. 2022;111:105276. doi:10.1016/j.nedt.2022.105276.

2 Suleiman-Martos N, Gomez-Urquiza JL, Aguayo-Estremera R, Cañadas-De La Fuente GA, De La Fuente-Solana EI, Albendín-García L. The effect of mindfulness training on burnout syndrome in nursing: A systematic review and meta-analysis. *J Adv Nurs*. 2020;76(5):1124–1140. doi:10.1111/jan.14318.
