# Supplementary material for: The relationship between self-efficacy, resilience, and job burnout in pediatric residents: a cross-sectional study in Western China
Source: BMC Med Educ. 2024 Jul 23;24:787. doi: 10.1186/s12909-024-05700-y (PMC11264473; doi:10.1186/s12909-024-05700-y)
Supplement: Supplementary file 1 — Supplementary Material 1 [file 12909_2024_5700_MOESM1_ESM.pdf]

Index	SubmitTime	CostTime	Gender	Educatior	Grade	Burnout_Q1	B_Q2	B_Q3	B_Q4	B_Q5	B_Q6	B_Q7	B_Q8	B_Q9	B_Q10	B_Q11	Resilience_Q1	R_Q2	R_Q3	R_Q4	R_Q5	R_Q6	R_Q7	R_Q8	R_Q9	R_Q10	Efficacy_Q1	E_Q2	E_Q3	E_Q4	E_Q5	E_Q6	E_Q7	E_Q8	E_Q9	E_Q10	Res	Effi	Burn1	Burn2	Burn3	Burn
1	2022/10/8 17:49:48	279秒	2	3	1	1	1	1	1	1	1	1	1	1	1	1	1	1	1	1	1	1	1	1	1	1	1	1	1	1	1	1	1	1	1	1	1.00	1.00	1.00	1.00	1.00	1.00
2	2022/10/8 17:51:08	366秒	2	4	3	4	4	5	3	3	5	4	5	2	3	2	4	4	4	4	3	3	3	3	3	4	3	3	2	2	2	3	2	2	3	2	3.50	2.40	3.80	4.67	2.33	3.64
3	2022/10/8 17:51:56	403秒	2	3	1	5	5	4	4	4	2	3	3	4	4	4	3	3	3	3	3	3	2	2	2	2	2	2	1	1	2	2	2	3	3	2	2.60	2.00	4.40	2.67	4.00	3.82
4	2022/10/8 17:52:12	422秒	2	3	1	1	2	4	4	4	1	1	1	1	1	1	4	4	5	5	4	4	4	4	4	4	3	3	3	3	3	3	3	3	3	3	4.20	3.00	3.00	1.00	1.00	1.91
5	2022/10/8 17:54:00	307秒	2	3	1	2	4	4	3	3	2	3	3	2	4	3	4	4	4	4	3	4	4	4	4	4	3	3	2	2	2	3	3	3	3	2	3.90	2.60	3.20	2.67	3.00	3.00
6	2022/10/8 17:54:02	204秒	1	4	1	4	4	4	4	4	4	4	4	4	4	4	3	3	3	3	3	3	3	3	3	3	3	3	3	3	3	3	3	3	3	3	3.00	3.00	4.00	4.00	4.00	4.00
7	2022/10/8 17:54:14	253秒	2	3	1	3	3	4	3	1	2	1	2	1	1	1	4	4	5	5	4	4	3	5	5	5	3	1	3	2	3	3	3	2	3	3	4.40	2.60	2.80	1.67	1.00	2.00
8	2022/10/8 17:55:05	351秒	2	2	3	4	4	3	3	3	3	3	4	2	3	4	4	4	3	5	4	4	3	4	4	4	3	2	1	3	1	4	2	2	3	1	3.90	2.20	3.40	3.33	3.00	3.27
9	2022/10/8 17:56:13	303秒	2	3	1	3	3	3	3	3	3	2	2	2	2	2	4	4	3	4	4	4	4	4	4	3	3	3	3	3	3	3	3	3	3	2	3.80	2.90	3.00	2.33	2.00	2.55
10	2022/10/8 17:56:42	454秒	2	3	1	2	2	3	2	2	2	2	2	2	2	1	4	4	4	4	5	4	4	4	4	4	3	2	2	3	3	3	3	3	3	3	4.10	2.80	2.20	2.00	1.67	2.00
11	2022/10/8 17:56:55	381秒	1	3	1	3	3	4	3	3	2	2	3	2	2	2	3	3	3	3	4	4	4	4	4	3	3	3	2	2	2	2	2	2	2	2	3.50	2.20	3.20	2.33	2.00	2.64
13	2022/10/8 17:57:28	317秒	2	3	1	3	4	4	3	3	3	3	3	3	3	3	4	3	4	3	4	3	3	3	3	3	2	2	1	1	1	1	1	1	1	1	3.30	1.20	3.40	3.00	3.00	3.18
14	2022/10/8 17:57:43	223秒	2	3	1	3	3	3	3	3	3	3	3	3	3	3	4	4	4	4	4	4	4	4	4	4	3	3	3	3	3	3	3	3	3	3	4.00	3.00	3.00	3.00	3.00	3.00
15	2022/10/8 17:58:05	346秒	2	2	2	4	4	4	4	3	2	3	4	2	2	2	3	3	3	3	3	3	3	3	3	3	3	2	2	2	2	3	3	2	3	3	3.00	2.50	3.80	3.00	2.00	3.09
16	2022/10/8 17:58:11	365秒	2	3	1	3	3	3	3	3	3	3	3	2	2	3	4	4	4	3	3	3	3	3	3	3	3	2	2	2	2	2	2	2	2	2	3.30	2.10	3.00	3.00	2.33	2.82
17	2022/10/8 17:59:13	836秒	1	3	1	2	3	3	3	2	2	2	2	2	2	2	4	4	4	4	4	4	4	4	4	4	3	2	2	3	2	3	3	3	3	2	4.00	2.60	2.60	2.00	2.00	2.27
19	2022/10/8 18:00:24	593秒	2	3	1	2	2	5	5	2	2	2	2	2	2	2	4	4	4	4	3	3	4	3	3	3	3	2	3	3	3	3	3	3	3	2	3.50	2.80	3.20	2.00	2.00	2.55
20	2022/10/8 18:00:32	275秒	2	3	1	3	3	5	4	3	3	3	3	3	2	2	3	3	4	4	4	4	4	4	4	4	3	3	3	3	3	3	3	3	3	3	3.80	3.00	3.60	3.00	2.33	3.09
21	2022/10/8 18:00:36	332秒	2	3	1	4	3	3	4	3	4	3	3	2	2	2	3	4	3	3	3	3	3	3	3	3	3	3	3	3	3	3	3	3	3	3	3.10	3.00	3.40	3.33	2.00	3.00
23	2022/10/8 18:01:09	250秒	2	3	1	4	4	4	4	3	2	4	4	4	3	4	4	4	4	3	3	3	2	3	3	2	2	2	2	2	2	2	3	3	3	3	3.10	2.40	3.80	3.33	3.67	3.64
24	2022/10/8 18:02:51	250秒	2	3	1	3	2	3	2	1	2	1	2	3	2	4	4	4	4	4	4	4	4	4	4	4	3	3	3	3	3	3	3	3	3	3	4.00	3.00	2.20	1.67	3.00	2.27
26	2022/10/8 18:03:05	441秒	2	3	1	2	2	4	4	3	2	1	3	2	2	2	4	4	4	4	4	4	3	4	4	4	4	3	2	2	2	3	3	3	3	2	3.90	2.70	3.00	2.00	2.00	2.45
27	2022/10/8 18:03:12	293秒	2	3	1	4	3	4	2	4	2	2	2	2	2	1	3	3	3	3	4	4	4	4	4	4	3	3	3	3	3	3	3	3	3	3	3.60	3.00	3.40	2.00	1.67	2.55
28	2022/10/8 18:03:27	455秒	2	3	1	3	4	4	3	3	3	3	2	2	3	3	4	3	3	3	4	3	3	3	3	3	2	2	2	2	2	2	2	2	2	2	3.20	2.00	3.40	2.67	2.67	3.00
29	2022/10/8 18:03:29	730秒	2	3	4	4	3	5	5	4	4	5	4	3	3	3	3	3	3	3	2	3	3	3	3	4	3	2	2	1	1	3	2	2	2	1	3.00	1.90	4.20	4.33	3.00	3.91
30	2022/10/8 18:03:51	492秒	2	3	1	5	5	5	5	5	5	5	3	3	2	2	4	4	4	4	4	4	4	4	4	4	3	3	3	3	3	3	3	3	3	3	4.00	3.00	5.00	4.33	2.33	4.09
32	2022/10/8 18:04:47	667秒	1	4	1	2	3	4	3	3	3	2	2	2	2	3	4	4	3	4	4	4	4	4	4	4	3	3	3	3	3	3	3	3	3	3	3.90	3.00	3.00	2.33	2.33	2.64
33	2022/10/8 18:04:51	694秒	1	2	4	4	4	4	4	3	2	4	2	2	2	2	4	4	5	5	4	4	3	5	5	4	4	4	2	3	2	3	3	3	3	3	4.30	3.00	3.80	2.67	2.00	3.00
34	2022/10/8 18:04:54	946秒	2	4	1	2	2	3	2	3	3	3	2	3	2	3	4	4	4	4	4	3	4	4	4	4	3	3	3	3	2	3	3	2	2	2	3.90	2.60	2.40	2.67	2.67	2.55
35	2022/10/8 18:06:24	912秒	2	3	1	3	3	3	3	2	2	2	3	2	2	2	3	3	3	3	3	3	4	3	4	3	3	2	2	2	2	3	2	2	2	2	3.20	2.20	2.80	2.33	2.00	2.45
36	2022/10/8 18:07:20	482秒	2	4	1	4	5	5	4	5	3	4	5	4	4	5	4	4	3	3	3	2	2	3	3	3	2	1	1	1	1	1	2	2	2	1	3.00	1.40	4.60	4.00	4.33	4.36
37	2022/10/8 18:08:01	531秒	2	3	1	4	5	5	5	4	4	4	3	3	3	3	4	3	3	2	2	2	3	3	3	3	2	1	1	1	1	2	2	2	2	1	2.80	1.50	4.60	3.67	3.00	3.91
39	2022/10/8 18:09:25	205秒	2	3	1	2	2	4	2	2	3	2	2	2	2	2	4	4	4	4	5	4	4	4	5	5	3	3	3	3	3	3	4	3	4	3	4.30	3.20	2.40	2.33	2.00	2.27
41	2022/10/8 18:12:32	266秒	2	3	1	3	3	3	3	3	3	3	3	3	3	3	4	4	4	4	4	4	4	4	4	4	3	3	3	3	3	3	3	3	3	3	4.00	3.00	3.00	3.00	3.00	3.00
42	2022/10/8 18:14:03	648秒	2	3	1	4	4	5	5	3	3	2	5	4	2	3	4	3	3	3	4	4	3	4	5	4	3	3	2	2	3	4	3	4	4	3	3.70	3.10	4.20	3.33	3.00	3.64
43	2022/10/8 18:14:27	302秒	1	3	1	4	5	5	5	5	1	2	2	1	3	3	4	4	4	4	2	2	3	2	3	3	3	3	3	3	2	3	3	3	3	2	3.10	2.80	4.80	1.67	2.33	3.27
44	2022/10/8 18:16:06	210秒	2	3	1	4	4	4	4	3	3	2	3	4	2	3	3	3	3	3	2	2	2	2	2	2	3	3	3	3	3	3	3	3	3	3	2.40	3.00	3.80	2.67	3.00	3.27
45	2022/10/8 18:18:32	313秒	2	3	1	3	3	4	4	3	3	4	4	2	3	3	3	3	3	3	3	3	3	3	3	3	3	3	3	3	3	3	3	3	3	3	3.00	3.00	3.40	3.67	2.67	3.27
48	2022/10/8 18:21:03	1274秒	2	3	1	2	4	4	4	4	2	2	2	2	3	2	4	3	4	4	4	4	4	4	4	4	3	3	3	3	3	3	3	3	3	3	3.90	3.00	3.60	2.00	2.33	2.82
49	2022/10/8 18:22:58	362秒	2	3	1	3	5	4	4	3	2	2	4	1	1	1	5	4	4	3	5	5	4	4	4	5	4	3	2	3	3	3	3	3	3	3	4.30	3.00	3.80	2.67	1.00	2.73
51	2022/10/8 18:24:30	451秒	2	4	1	2	2	4	3	3	2	2	2	2	4	2	4	4	4	3	4	3	3	3	3	3	3	3	2	2	2	2	2	2	2	2	3.40	2.20	2.80	2.00	2.67	2.55
52	2022/10/8 18:27:59	833秒	2	3	1	2	2	3	2	2	2	2	2	2	2	3	4	4	4	4	4	4	4	4	4	4	3	3	3	3	3	3	3	2	3	3	4.00	2.90	2.20	2.00	2.33	2.18
53	2022/10/8 18:29:19	2067秒	2	4	1	4	4	4	4	4	3	3	4	3	2	2	4	4	4	3	4	4	3	4	4	4	3	3	2	2	3	3	3	3	4	3	3.80	2.90	4.00	3.33	2.33	3.36
54	2022/10/8 18:30:22	932秒	2	4	1	5	5	4	5	3	2	2	5	4	3	5	3	3	4	4	4	3	2	2	2	3	3	2	1	1	1	4	2	3	3	2	3.00	2.20	4.40	3.00	4.00	3.91
55	2022/10/8 18:37:39	363秒	2	4	1	4	4	5	4	3	3	3	2	4	2	3	4	4	3	3	4	4	4	3	4	4	3	2	2	3	3	3	3	3	3	3	3.70	2.80	4.00	2.67	3.00	3.36
56	2022/10/8 18:38:29	641秒	2	3	1	3	3	4	3	3	4	3	2	3	2	3	4	4	4	3	4	4	3	4	4	4	3	3	2	3	2	3	3	3	3	2	3.80	2.70	3.20	3.00	2.67	3.00
57	2022/10/8 18:42:27	551秒	2	3	1	4	4	4	3	3	3	4	4	4	3	3	4	4	3	4	4	4	2	3	3	3	3	2	2	3	2	4	2	2	2	2	3.40	2.40	3.60	3.67	3.33	3.55
58	2022/10/8 18:44:48	169秒	1	3	4	3	3	3	3	3	3	3	3	3	3	3	3	3	3	3	3	3	3	3	3	3	3	3	3	3	3	3	3	3	3	3	3.00	3.00	3.00	3.00	3.00	3.00
59	2022/10/8 18:45:19	149秒	1	4	1	3	3	3	3	3	3	3	3	3	3	3	3	3	3	3	3	3	3	3	3	3	2	2	2	2	2	2	2	2	2	2	3.00	2.00	3.00	3.00	3.00	3.00
60	2022/10/8 18:45:34	238秒	1	4	1	2	2	3	2	2	2	2	2	2	2	2	3	3	3	3	3	3	3	3	3	3	3	2	2	2	2	2	2	2	2	2	3.00	2.10	2.20	2.00	2.00	2.09
61	2022/10/8 18:46:13	743秒	2	3	1	4	4	4	5	4	3	2	2	2	2	2	4	4	4	4	4	4	4	4	4	4	3	3	3	3	3	3	3	3	3	3	4.00	3.00	4.20	2.33	2.00	3.09
62	2022/10/8 18:46:23	245秒	2	3	2	1	5	5	5	5	5	5	1	3	5	3	4	2	3	4	4	4	2	4	4	1	3	2	2	1	2	2	2	2	2	2	3.20	2.00	4.20	3.67	3.67	3.91
63	2022/10/8 18:47:22	476秒	2	4	1	3	4	5	4	3	3	2	3	2	2	3	3	3	3	3	3	3	4	4	3	3	2	2	2	2	2	3	2	2	3	2	3.20	2.20	3.80	2.67	2.33	3.09
65	2022/10/8 18:48:34	287秒	2	3	2	3	3	3	3	3	3	3	3	3	3	3	3	3	3	3	3	3	3	3	3	3	3	2	1	1	2	2	1	1	2	1	3.00	1.60	3.00	3.00	3.00	3.00
66	2022/10/8 18:49:05	555秒	2	2	4	3	4	4	4	4	4	4	4	3	4	4	4	3	3	4	4	4	3	3	4	4	3	3	3	3	2	3	3	3	3	3	3.60	2.90	3.80	4.00	3.67	3.82
67	2022/10/8 18:49:13	374秒	2	3	2	3	4	5	4	4	3	4	5	3	3	2	3	3	3	4	3	3	3	4	5	4	3	1	1	1	1	2	2	2	2	2	3.50	1.70	4.00	4.00	2.67	3.64
68	2022/10/8 18:51:03	761秒	2	3	1	4	5	5	5	4	3	3	3	4	2	5	4	4	4	3	4	4	4	4	4	4	3	3	3	3	3	3	3	3	3	3	3.90	3.00	4.60	3.00	3.67	3.91
69	2022/10/8 18:52:13	1082秒	2	3	1	4	5	5	5	4	1	3	2	3	3	4	5	5	5	5	4	4	3	4	4	5	3	3	3	1	2	3	3	3	3	2	4.40	2.60	4.60	2.00	3.33	3.55
70	2022/10/8 18:53:16	2652秒	2	2	4	3	3	3	3	3	4	3	4	3	3	3	4	3	3	3	3	3	3	3	3	3	3	2	2	2	2	2	2	2	2	2	3.10	2.10	3.00	3.67	3.00	3.18
71	2022/10/8 18:53:19	405秒	2	3	2	2	3	5	5	3	3	3	3	3	4	3	3	3	3	2	2	2	2	2	2	2	2	1	2	1	2	2	2	2	2	2	2.30	1.80	3.60	3.00	3.33	3.36
72	2022/10/8 18:55:31	656秒	2	3	1	2	3	3	2	3	3	2	2	2	2	3	4	4	4	5	4	4	4	4	5	5	3	3	2	2	2	2	2	2	2	2	4.30	2.20	2.60	2.33	2.33	2.45
73	2022/10/8 18:56:20	353秒	1	3	1	3	4	5	3	4	3	3	4	4	4	2	3	3	4	1	4	3	3	2	4	3	3	3	1	1	2	4	3	2	2	1	3.00	2.20	3.80	3.33	3.33	3.55
75	2022/10/8 18:58:02	738秒	2	3	1	4	4	4	4	4	3	3	2	3	4	4	3	3	3	3	3	3	3	3	3	3	3	3	2	2	2	2	2	2	2	2	3.00	2.20	4.00	2.67	3.67	3.55
76	2022/10/8 19:03:45	578秒	1	3	1	3	3	3	3	3	3	2	2	3	2	3	4	4	4	4	4	4	4	4	4	4	3	3	2	3	2	2	3	3	3	3	4.00	2.70	3.00	2.33	2.67	2.73
77	2022/10/8 19:04:12	186秒	1	2	2	4	4	5	4	2	3	3	3	2	2	2	4	4	4	4	4	4	4	4	4	4	3	3	3	3	3	3	3	3	3	3	4.00	3.00	3.80	3.00	2.00	3.09
78	2022/10/8 19:06:39	404秒	2	4	1	2	3	5	3	2	3	2	2	2	3	2	4	3	4	4	4	4	4	4	4	4	3	3	3	3	3	3	3	2	3	3	3.90	2.90	3.00	2.33	2.33	2.64
80	2022/10/8 19:07:30	132秒	2	4	2	5	5	5	5	5	5	5	5	5	5	5	2	2	2	2	2	2	2	2	2	2	2	2	2	2	2	2	2	2	2	2	2.00	2.00	5.00	5.00	5.00	5.00
81	2022/10/8 19:10:15	299秒	2	3	1	4	4	4	3	3	3	3	3	3	3	3	3	3	3	3	3	3	3	3	3	3	2	2	2	2	2	2	2	2	2	2	3.00	2.00	3.60	3.00	3.00	3.27
82	2022/10/8 19:12:39	815秒	2	3	1	2	2	3	2	1	2	2	2	1	1	1	5	5	4	4	1	4	4	5	4	4	3	2	1	3	3	3	3	3	3	3	4.00	2.70	2.00	2.00	1.00	1.73
83	2022/10/8 19:14:27	1796秒	2	2	3	5	5	5	5	5	3	5	5	5	5	5	4	3	4	3	3	2	2	3	2	4	2	3	2	2	2	3	2	3	2	1	3.00	2.20	5.00	4.33	5.00	4.82
84	2022/10/8 19:18:20	911秒	2	3	1	2	2	3	3	3	3	2	3	1	1	1	3	3	3	3	3	3	3	3	3	3	2	2	2	2	2	2	2	2	2	2	3.00	2.00	2.60	2.67	1.00	2.18
85	2022/10/8 19:18:32	440秒	2	3	1	3	5	4	4	3	3	2	4	3	2	3	3	3	3	3	3	3	3	3	3	3	3	2	1	2	2	3	2	1	2	1	3.00	1.90	3.80	3.00	2.67	3.27
86	2022/10/8 19:20:35	523秒	2	4	1	3	3	3	3	3	3	3	3	3	3	3	3	3	3	3	3	3	3	3	3	3	3	2	2	2	2	2	3	2	2	2	3.00	2.20	3.00	3.00	3.00	3.00
87	2022/10/8 19:27:58	346秒	2	3	1	2	3	3	3	2	3	3	1	2	3	3	3	3	3	3	3	3	3	3	3	3	2	2	2	2	2	2	2	2	2	2	3.00	2.00	2.60	2.33	2.67	2.55
88	2022/10/8 19:31:09	290秒	1	3	1	4	4	4	2	2	1	1	1	2	1	1	5	5	5	5	5	5	5	5	5	5	4	3	3	3	3	4	4	4	4	3	5.00	3.50	3.20	1.00	1.33	2.09
89	2022/10/8 19:51:49	334秒	2	3	1	3	2	3	2	2	3	3	2	3	3	3	4	4	4	4	2	4	4	4	4	4	3	3	2	3	2	3	3	3	3	3	3.80	2.80	2.40	2.67	3.00	2.64
91	2022/10/8 20:02:19	162秒	2	2	3	3	3	3	3	3	3	3	3	3	3	3	4	4	4	4	4	4	4	4	4	4	3	3	3	3	3	3	3	3	3	3	4.00	3.00	3.00	3.00	3.00	3.00

92	2022/10/8 20:05:02	1032秒	2	4	1	4	4	4	4	3	2	4	4	2	2	3	4	4	4	4	4	4	4	4	4	4	3	3	2	3	3	3	3	3	3	3	4.00	2.90	3.80	3.33	2.33	3.27
93	2022/10/8 20:07:28	867秒	2	3	1	3	3	4	3	3	3	2	2	2	2	2	4	4	4	4	4	4	3	4	4	4	3	3	3	3	3	4	4	3	3	3	3.90	3.20	3.20	2.33	2.00	2.64
95	2022/10/8 20:29:58	824秒	1	2	1	2	3	4	2	3	2	1	2	2	2	2	4	4	4	4	4	4	4	4	4	4	3	3	2	3	3	3	3	2	3	2	4.00	2.70	2.80	1.67	2.00	2.27
96	2022/10/8 20:37:32	713秒	2	3	1	2	4	4	3	3	1	2	1	2	2	1	4	4	3	3	4	4	3	4	4	4	3	2	2	2	2	2	2	2	2	2	3.70	2.10	3.20	1.33	1.67	2.27
97	2022/10/8 20:44:21	691秒	2	2	2	3	3	4	4	3	3	3	3	3	3	3	3	3	3	3	3	3	3	3	3	3	3	3	2	2	2	3	2	3	3	2	3.00	2.50	3.40	3.00	3.00	3.18
98	2022/10/8 20:49:38	213秒	2	3	1	3	3	3	3	3	3	3	3	1	1	1	4	4	4	4	4	4	4	4	4	4	3	3	3	3	3	3	3	3	3	3	4.00	3.00	3.00	3.00	1.00	2.45
99	2022/10/8 20:49:46	539秒	2	4	1	4	4	4	4	4	4	4	4	4	4	3	3	3	3	3	3	3	3	3	3	3	2	2	2	2	2	2	1	2	2	1	3.00	1.80	4.00	4.00	3.67	3.91
100	2022/10/8 21:30:56	448秒	1	3	1	2	2	3	1	1	2	2	2	2	2	2	4	4	4	4	4	4	4	4	4	4	3	3	3	3	3	3	3	3	3	3	4.00	3.00	1.80	2.00	2.00	1.91
101	2022/10/8 21:51:35	791秒	2	2	3	4	4	3	2	4	3	4	3	2	2	3	3	3	3	3	3	3	3	4	3	3	3	2	3	2	2	3	2	2	2	2	3.10	2.30	3.40	3.33	2.33	3.09
102	2022/10/8 22:11:55	1328秒	2	2	3	3	3	4	3	3	2	2	2	2	2	2	3	3	3	3	2	3	3	3	3	2	3	2	2	2	2	2	2	2	2	2	2.80	2.10	3.20	2.00	2.00	2.55
103	2022/10/8 22:16:33	373秒	2	3	1	3	4	4	3	3	4	4	3	3	3	3	4	4	4	4	4	4	4	4	4	4	3	3	3	3	3	3	3	3	3	3	4.00	3.00	3.40	3.67	3.00	3.36
104	2022/10/8 22:31:20	220秒	2	4	1	3	4	5	4	4	3	3	2	2	1	1	4	4	4	4	4	4	4	4	4	4	3	3	3	3	3	3	3	3	3	3	4.00	3.00	4.00	2.67	1.33	2.91
106	2022/10/8 23:17:14	755秒	2	2	2	4	5	5	5	3	3	3	3	3	3	3	4	3	4	3	3	3	3	3	3	3	2	2	2	2	2	2	2	2	2	2	3.20	2.00	4.40	3.00	3.00	3.64
107	2022/10/8 23:41:58	398秒	2	3	1	2	3	3	3	2	3	3	3	2	2	2	3	3	4	3	3	3	3	3	3	3	3	2	2	2	2	3	2	2	2	2	3.10	2.20	2.60	3.00	2.00	2.55
110	2022/10/9 8:31:59	258秒	2	3	1	2	3	5	4	3	3	2	2	2	2	2	4	4	4	3	4	4	3	4	4	4	3	3	2	2	3	3	3	3	3	2	3.80	2.70	3.40	2.33	2.00	2.73
112	2022/10/9 8:43:36	194秒	2	3	1	3	3	3	2	2	3	3	3	3	2	2	4	4	4	4	4	4	4	4	4	4	3	3	3	3	2	3	2	2	2	2	4.00	2.50	2.60	3.00	2.33	2.64
113	2022/10/9 8:44:24	252秒	1	3	2	3	3	3	3	3	3	3	3	3	3	3	3	3	3	3	5	5	5	5	5	5	4	4	4	4	4	4	4	4	4	4	4.20	4.00	3.00	3.00	3.00	3.00
114	2022/10/9 8:46:04	1327秒	2	4	4	3	3	4	4	2	2	2	2	2	2	3	4	3	4	3	3	4	4	4	4	4	3	2	2	2	2	3	3	2	3	2	3.70	2.40	3.20	2.00	2.33	2.64
116	2022/10/9 8:54:07	394秒	2	3	2	3	3	3	3	3	3	3	3	3	3	3	3	3	3	3	3	3	3	3	3	3	2	2	2	2	2	2	2	2	2	2	3.00	2.00	3.00	3.00	3.00	3.00
117	2022/10/9 8:54:47	401秒	2	4	1	4	4	4	4	3	3	3	3	3	3	4	4	4	4	4	3	4	4	4	4	4	2	2	2	2	2	2	2	2	2	2	3.90	2.00	3.80	3.00	3.33	3.45
118	2022/10/9 8:55:41	363秒	2	2	3	3	3	3	3	3	3	3	3	3	3	3	3	3	3	3	3	3	3	3	3	3	2	2	2	2	2	2	2	2	2	2	3.00	2.00	3.00	3.00	3.00	3.00
119	2022/10/9 8:57:30	706秒	2	2	2	4	5	5	5	5	4	5	5	3	3	2	3	4	4	4	4	4	4	4	4	4	3	3	2	2	2	2	1	1	2	2	3.90	2.00	4.80	4.67	2.67	4.18
120	2022/10/9 8:59:33	431秒	1	2	4	2	2	3	2	3	2	2	3	2	2	2	3	3	4	3	4	3	3	4	4	4	3	3	2	2	2	3	3	2	3	3	3.50	2.60	2.40	2.33	2.00	2.27
121	2022/10/9 9:00:04	238秒	2	2	2	4	4	4	3	4	2	3	3	3	3	3	3	3	3	3	3	3	3	3	3	3	3	2	2	2	2	2	2	2	2	2	3.00	2.10	3.80	2.67	3.00	3.27
122	2022/10/9 9:02:14	1543秒	1	3	1	3	3	3	1	3	3	3	3	1	2	1	3	3	3	3	3	3	3	3	3	3	3	2	2	2	2	2	2	2	2	2	3.00	2.10	2.60	3.00	1.33	2.36
124	2022/10/9 9:03:33	364秒	2	3	1	3	3	4	4	2	1	1	1	1	1	2	4	3	3	3	3	3	3	3	3	3	2	2	2	2	1	3	2	2	2	2	3.10	2.00	3.20	1.00	1.33	2.09
125	2022/10/9 9:08:53	453秒	2	2	3	4	4	5	4	3	4	4	5	3	2	2	4	4	4	4	4	4	4	4	4	3	3	3	2	3	2	3	3	2	3	3	3.90	2.70	4.00	4.33	2.33	3.64
126	2022/10/9 9:28:16	249秒	2	2	2	2	2	3	2	3	3	2	3	2	3	2	4	4	4	4	4	4	4	4	4	4	3	3	3	3	3	3	3	3	3	3	4.00	3.00	2.40	2.67	2.33	2.45
127	2022/10/9 9:28:22	317秒	2	2	4	4	4	4	3	4	2	3	2	2	2	3	4	4	4	3	4	4	4	4	4	3	3	3	2	2	2	2	2	3	3	2	3.80	2.40	3.80	2.33	2.33	3.00
128	2022/10/9 9:28:49	2847秒	2	3	1	2	3	3	2	3	2	2	3	2	2	2	3	3	4	4	3	3	3	4	4	4	3	3	2	2	2	3	2	2	2	2	3.50	2.30	2.60	2.33	2.00	2.36
129	2022/10/9 9:36:34	265秒	2	3	1	1	1	2	2	2	3	3	2	1	1	1	5	3	3	4	4	4	2	3	3	3	4	3	2	2	2	2	2	3	3	2	3.40	2.50	1.60	2.67	1.00	1.73
130	2022/10/9 9:38:19	1241秒	2	2	4	4	4	4	4	4	4	4	4	4	4	5	4	4	3	3	3	3	3	3	3	3	3	2	2	2	2	3	2	2	2	2	3.20	2.20	4.00	4.00	4.33	4.09
131	2022/10/9 9:49:22	1605秒	2	2	4	3	4	4	3	3	4	3	3	3	2	2	4	4	4	4	3	4	3	4	4	4	3	2	1	2	1	3	3	2	2	1	3.80	2.00	3.40	3.33	2.33	3.09
132	2022/10/9 9:50:53	256秒	2	3	3	5	5	5	4	3	3	3	3	3	3	3	3	3	3	3	3	3	3	3	3	3	2	2	2	2	2	2	2	2	2	2	3.00	2.00	4.40	3.00	3.00	3.64
133	2022/10/9 10:03:33	556秒	1	2	3	3	2	3	3	2	3	2	2	2	2	2	3	4	4	4	3	3	3	4	4	4	3	3	3	3	3	3	3	3	3	3	3.60	3.00	2.60	2.33	2.00	2.36
134	2022/10/9 10:07:55	835秒	2	3	1	3	3	3	2	2	2	2	3	2	2	3	5	4	4	4	4	4	3	4	3	4	3	3	2	2	2	3	3	2	2	2	3.90	2.40	2.60	2.33	2.33	2.45
135	2022/10/9 10:10:20	691秒	2	2	4	4	4	4	4	2	2	2	2	2	1	1	5	5	5	4	4	5	5	5	5	5	4	3	3	3	3	3	3	3	3	3	4.80	3.10	3.60	2.00	1.33	2.55
136	2022/10/9 10:15:38	173秒	2	2	2	2	3	4	4	4	3	2	3	2	3	2	3	3	3	4	4	4	4	4	4	4	3	2	2	3	2	3	3	1	2	2	3.70	2.30	3.40	2.67	2.33	2.91
137	2022/10/9 10:16:40	926秒	2	3	1	2	2	4	2	2	3	2	2	2	2	2	4	4	4	4	4	4	4	4	4	4	3	2	1	2	2	3	3	3	3	2	4.00	2.40	2.40	2.33	2.00	2.27
138	2022/10/9 10:17:27	511秒	1	2	3	1	1	1	1	1	1	1	1	1	1	1	4	4	4	3	4	3	4	4	5	5	3	2	1	2	1	2	2	2	2	2	4.00	1.90	1.00	1.00	1.00	1.00
139	2022/10/9 10:41:52	698秒	2	3	4	3	4	5	5	4	3	3	3	3	3	3	3	3	3	3	3	3	3	3	3	3	2	2	2	1	1	2	1	1	2	1	3.00	1.50	4.20	3.00	3.00	3.55
140	2022/10/9 11:27:39	713秒	2	3	4	5	5	5	5	5	5	5	5	5	5	5	4	4	2	2	2	2	2	4	4	4	3	3	3	3	3	3	3	3	3	3	3.00	3.00	5.00	5.00	5.00	5.00
141	2022/10/9 11:28:23	287秒	1	2	4	5	5	5	5	5	4	5	1	1	1	1	5	5	5	5	1	5	5	5	5	5	4	3	3	3	3	3	3	3	3	3	4.60	3.10	5.00	3.33	1.00	3.45
142	2022/10/9 12:13:31	404秒	2	2	3	3	2	3	2	3	4	3	4	1	1	1	4	4	3	4	4	3	2	4	4	3	3	2	2	2	2	3	3	3	3	2	3.50	2.50	2.60	3.67	1.00	2.45
143	2022/10/9 12:17:41	177秒	2	4	1	3	3	3	3	3	3	3	3	3	3	3	4	4	4	4	4	4	4	4	4	4	3	3	3	3	3	3	3	3	3	3	4.00	3.00	3.00	3.00	3.00	3.00
144	2022/10/9 12:24:08	736秒	2	4	1	3	3	4	4	3	2	2	2	2	3	3	4	4	3	3	3	4	4	4	4	4	3	2	2	2	2	3	3	3	3	3	3.70	2.60	3.40	2.00	2.67	2.82
145	2022/10/9 12:38:09	541秒	2	2	4	3	4	5	4	3	4	3	3	3	3	3	3	3	3	3	3	3	3	3	3	3	2	2	2	2	1	2	2	2	2	1	3.00	1.80	3.80	3.33	3.00	3.45
146	2022/10/9 13:29:28	249秒	2	2	2	3	3	3	3	3	3	3	3	3	3	3	3	3	3	3	3	3	3	3	3	3	3	2	2	1	1	3	2	2	2	2	3.00	2.00	3.00	3.00	3.00	3.00
147	2022/10/9 15:10:46	417秒	2	3	1	3	3	3	2	2	2	2	2	3	2	2	4	4	4	4	2	2	2	2	2	2	3	3	3	3	2	2	2	2	2	2	2.80	2.40	2.60	2.00	2.33	2.36
148	2022/10/9 16:10:44	217秒	2	2	2	3	2	3	2	2	2	2	2	2	1	2	3	2	2	2	2	3	2	2	2	2	3	3	3	3	3	3	3	3	3	3	2.20	3.00	2.40	2.00	1.67	2.09
149	2022/10/9 17:04:01	217秒	2	3	3	3	3	3	4	3	4	3	4	2	2	2	4	4	4	4	4	4	4	4	4	4	3	3	3	3	3	3	3	3	3	3	4.00	3.00	3.20	3.67	2.00	3.00
150	2022/10/9 17:04:07	235秒	2	3	2	3	3	4	3	4	3	4	4	3	3	4	3	3	3	3	3	3	4	3	3	3	2	2	1	2	2	1	1	1	2	1	3.10	1.50	3.40	3.67	3.33	3.45
151	2022/10/9 17:04:10	197秒	2	3	3	3	3	4	4	3	3	3	3	3	3	2	5	5	5	5	5	5	5	5	5	5	4	4	4	4	4	4	4	4	4	4	5.00	4.00	3.40	3.00	2.67	3.09
152	2022/10/9 17:04:33	240秒	2	3	4	3	3	4	2	1	2	2	1	1	1	1	4	4	4	4	4	4	4	4	3	4	3	3	2	3	3	3	3	3	3	2	3.90	2.80	2.60	1.67	1.00	1.91
154	2022/10/9 17:05:54	337秒	2	3	3	4	4	4	4	3	3	4	3	3	3	3	4	3	4	3	3	3	3	3	3	3	3	1	1	1	1	2	2	2	3	1	3.20	1.70	3.80	3.33	3.00	3.45
155	2022/10/9 17:06:30	369秒	2	3	4	3	3	4	3	3	4	3	4	3	3	3	3	3	3	3	3	3	3	3	3	3	3	3	3	3	3	3	3	3	3	3	3.00	3.00	3.20	3.67	3.00	3.27
156	2022/10/9 17:07:12	382秒	2	3	1	3	4	4	4	4	3	3	2	2	1	1	3	3	3	3	3	3	3	2	2	3	2	2	2	2	2	1	2	2	2	2	2.80	1.90	3.80	2.67	1.33	2.82
157	2022/10/9 17:07:50	163秒	2	3	4	3	3	3	3	3	3	3	3	3	3	3	5	3	3	3	3	3	3	3	3	3	3	3	3	3	3	3	3	3	3	3	3.20	3.00	3.00	3.00	3.00	3.00
158	2022/10/9 17:08:56	127秒	2	2	3	4	4	4	4	3	4	3	3	3	3	3	4	4	4	4	4	4	4	4	4	4	3	3	3	3	3	3	3	3	3	3	4.00	3.00	3.80	3.33	3.00	3.45
159	2022/10/9 17:09:29	313秒	1	2	3	1	1	1	1	1	1	1	1	1	1	1	4	4	4	4	4	4	4	4	4	4	3	3	3	3	3	3	3	3	3	3	4.00	3.00	1.00	1.00	1.00	1.00
160	2022/10/9 17:10:57	128秒	1	2	2	5	5	5	5	5	5	5	5	5	5	5	1	1	1	1	1	1	1	1	1	1	1	1	1	1	1	1	1	1	1	1	1.00	1.00	5.00	5.00	5.00	5.00
161	2022/10/9 17:12:02	772秒	2	2	2	2	2	5	4	2	2	1	1	1	4	1	4	4	4	4	5	5	5	5	5	5	3	3	3	4	3	4	3	4	4	3	4.60	3.40	3.00	1.33	2.00	2.27
162	2022/10/9 17:12:43	333秒	1	2	2	2	2	4	3	2	2	1	3	2	3	3	3	2	2	3	2	3	2	4	4	4	3	2	2	2	1	2	3	3	3	2	2.90	2.30	2.60	2.00	2.67	2.45
163	2022/10/9 17:17:13	966秒	2	3	3	5	5	5	5	5	4	5	5	4	4	4	4	4	4	3	3	3	2	3	3	3	3	3	2	2	2	3	2	2	2	2	3.20	2.30	5.00	4.67	4.00	4.64
164	2022/10/9 17:17:55	490秒	1	2	2	2	2	5	4	3	2	1	3	3	4	2	4	4	4	4	4	5	4	4	5	5	3	2	2	2	2	3	4	3	3	3	4.30	2.70	3.20	2.00	3.00	2.82
165	2022/10/9 17:19:42	637秒	2	3	2	5	5	5	5	5	3	5	3	4	3	4	3	3	2	2	2	3	2	3	3	3	4	3	1	2	1	2	1	1	2	1	2.60	1.80	5.00	3.67	3.67	4.27
166	2022/10/9 17:22:32	345秒	2	2	2	4	4	4	4	4	5	4	3	4	4	4	3	2	3	2	3	2	3	2	3	2	2	1	1	1	1	1	1	1	1	1	2.50	1.10	4.00	4.00	4.00	4.00
167	2022/10/9 17:25:19	299秒	2	2	2	3	3	5	5	4	3	3	3	3	3	3	3	3	3	3	3	3	3	3	3	3	3	2	2	2	2	2	3	2	2	2	3.00	2.20	4.00	3.00	3.00	3.45
168	2022/10/9 17:25:50	566秒	2	3	1	3	4	4	4	4	4	3	3	3	2	3	4	3	3	3	3	4	3	3	3	3	3	3	1	2	2	2	2	3	3	2	3.20	2.30	3.80	3.33	2.67	3.36
169	2022/10/9 17:31:34	914秒	1	2	2	5	5	5	5	4	3	3	3	3	3	3	2	2	2	2	3	3	3	3	3	2	2	2	3	3	2	2	2	2	2	2	2.50	2.20	4.80	3.00	3.00	3.82
170	2022/10/9 17:33:17	372秒	2	3	2	3	3	3	1	4	3	1	5	2	1	1	3	3	4	5	1	4	1	5	5	5	4	4	1	2	2	3	4	3	4	1	3.60	2.80	2.80	3.00	1.33	2.45
171	2022/10/9 17:40:11	349秒	2	2	3	2	3	5	4	2	3	2	2	2	2	2	5	4	4	4	3	4	3	4	4	4	3	2	1	2	1	3	3	2	2	2	3.90	2.10	3.20	2.33	2.00	2.64
172	2022/10/9 17:57:46	681秒	1	2	3	3	2	3	3	1	1	2	1	1	1	1	5	4	4	5	5	5	5	5	5	5	4	4	3	3	4	4	3	3	3	3	4.80	3.40	2.40	1.33	1.00	1.73
173	2022/10/9 18:02:11	783秒	2	2	4	2	4	4	4	4	4	2	5	3	2	4	3	4	4	4	3	3	3	3	4	4	3	3	2	2	2	3	3	3	3	2	3.50	2.60	3.60	3.67	3.00	3.45
174	2022/10/9 18:09:33	440秒	2	3	3	5	5	5	5	5	5	3	5	5	5	5	2	2	2	2	2	2	2	2	2	2	2	1	1	1	1	1	1	1	1	1	2.00	1.10	5.00	4.33	5.00	4.82
175	2022/10/9 18:22:58	568秒	2	4	1	3	4	4	4	3	3	3	4	3	3	3	4	3	4	3	3	3	3	3	3	3	3	2	2	2	2	3	3	2	3	2	3.20	2.40	3.60	3.33	3.00	3.36
176	2022/10/9 18:27:42	1127秒	2	3	2	3	3	5	4	2	2	2	4	3	3	3	3	3	3	3	3	3	3	3	3	3	2	2	2	2	2	2	2	2	2	2	3.00	2.00	3.40	2.67	3.00	3.09
177	2022/10/9 18:48:31	109秒	2	3	2	3	3	3	3	3	3	3	3	3	3	3	3	3	3	3	3	3	3	3	3	3	3	2	3	2	3	2	2	2	3	2	3.00	2.40	3.00	3.00	3.00	3.00

178	2022/10/9 19:19:06	842秒	2	2	2	3	5	5	5	5	1	5	3	3	3	3	3	3	3	3	2	3	2	3	5	3	3	3	3	3	3	3	3	3	3	3	3.00	3.00	4.60	3.00	3.00	3.73
179	2022/10/9 19:20:37	562秒	2	2	3	3	5	4	4	4	3	3	4	3	2	3	4	3	4	4	4	4	3	4	3	4	3	2	2	2	3	3	2	3	2	2	3.70	2.40	4.00	3.33	2.67	3.45
180	2022/10/9 19:27:12	223秒	1	3	2	1	1	1	1	1	1	1	1	1	1	1	5	5	5	5	5	5	5	5	5	5	4	4	4	4	4	4	4	4	4	4	5.00	4.00	1.00	1.00	1.00	1.00
181	2022/10/9 20:02:37	1333秒	2	2	4	4	5	5	5	4	4	3	5	2	3	3	5	3	5	3	4	3	4	4	3	4	3	3	2	3	2	3	2	3	3	2	3.80	2.60	4.60	4.00	2.67	3.91
182	2022/10/9 21:21:12	327秒	1	2	2	5	4	4	4	1	3	4	3	4	4	3	4	4	5	5	4	4	4	4	4	4	3	3	3	3	2	3	3	3	3	3	4.20	2.90	3.60	3.33	3.67	3.55
183	2022/10/9 22:05:44	1583秒	2	2	4	4	5	5	4	4	3	4	4	4	3	3	4	3	2	3	2	3	3	3	3	3	2	2	2	2	2	1	2	2	2	2	2.90	1.90	4.40	3.67	3.33	3.91
184	2022/10/9 22:17:04	731秒	2	2	4	2	5	5	5	4	4	2	2	2	3	2	4	3	2	2	3	3	2	3	2	3	2	3	2	2	2	3	2	3	3	2	2.70	2.40	4.20	2.67	2.33	3.27
186	2022/10/10 1:53:35	330秒	1	2	4	3	3	3	3	3	3	3	3	2	2	4	4	4	4	4	5	5	4	4	4	4	3	2	2	2	2	3	2	2	3	3	4.20	2.40	3.00	3.00	2.67	2.91
187	2022/10/10 8:21:00	928秒	2	2	4	3	3	4	3	3	3	2	2	3	2	3	3	3	3	3	3	3	3	3	3	3	3	2	2	1	1	3	2	2	2	1	3.00	1.90	3.20	2.33	2.67	2.82
188	2022/10/10 8:24:25	344秒	2	2	4	3	4	4	3	3	4	4	4	3	3	4	2	3	3	3	3	3	3	3	2	2	2	2	2	2	2	2	2	2	2	2	2.70	2.00	3.40	4.00	3.33	3.55
189	2022/10/10 8:28:50	652秒	2	3	1	1	1	4	3	2	2	1	1	1	1	2	4	4	4	3	3	3	3	4	4	4	3	2	3	2	2	2	3	2	3	2	3.60	2.40	2.20	1.33	1.33	1.73
190	2022/10/10 8:46:21	295秒	2	3	2	2	4	5	4	3	3	3	3	3	2	4	3	3	4	3	3	3	3	4	4	4	2	2	1	2	2	2	2	2	2	2	3.40	1.90	3.60	3.00	3.00	3.27
191	2022/10/10 8:50:26	1289秒	2	2	4	2	3	5	3	5	4	3	3	5	5	4	2	2	4	3	2	2	3	2	3	2	2	2	2	1	1	2	2	1	2	1	2.50	1.60	3.60	3.33	4.67	3.82
192	2022/10/10 8:53:00	319秒	2	3	2	4	3	4	4	4	3	3	3	2	3	3	4	4	4	4	4	4	4	4	4	3	3	3	3	3	3	3	3	3	3	3	3.90	3.00	3.80	3.00	2.67	3.27
193	2022/10/10 8:56:32	390秒	1	3	2	4	4	5	5	5	4	4	4	4	3	4	3	3	3	3	3	3	3	3	3	3	2	2	1	1	2	2	2	2	2	2	3.00	1.80	4.60	4.00	3.67	4.18
194	2022/10/10 9:19:41	774秒	2	3	1	3	3	3	3	3	3	3	3	1	2	1	4	4	4	4	4	4	4	4	4	4	3	3	2	3	3	3	3	3	3	3	4.00	2.90	3.00	3.00	1.33	2.55
195	2022/10/10 9:22:09	164秒	2	2	2	3	3	3	3	3	3	3	3	3	3	3	3	3	3	3	3	3	3	3	3	3	3	3	3	3	3	3	3	3	3	3	3.00	3.00	3.00	3.00	3.00	3.00
196	2022/10/10 9:23:31	1604秒	2	4	1	3	3	4	4	3	4	3	3	2	2	2	3	3	4	4	3	3	3	3	3	3	2	1	2	1	2	2	2	1	2	1	3.20	1.60	3.40	3.33	2.00	3.00
197	2022/10/10 9:30:58	1292秒	2	2	3	5	5	5	5	5	5	5	5	5	5	5	2	2	2	2	2	2	2	2	2	2	2	2	2	2	2	2	2	2	2	2	2.00	2.00	5.00	5.00	5.00	5.00
198	2022/10/10 9:32:29	491秒	2	2	4	3	4	4	4	3	3	3	4	3	3	2	3	3	3	3	3	3	3	3	4	4	3	2	2	2	2	3	2	2	2	2	3.20	2.20	3.60	3.33	2.67	3.27
199	2022/10/10 9:34:38	344秒	2	2	3	3	3	4	3	3	4	3	3	2	3	3	3	3	4	3	3	3	3	3	3	3	2	2	2	2	2	2	2	2	2	2	3.10	2.00	3.20	3.33	2.67	3.09
200	2022/10/10 9:47:21	350秒	2	3	4	5	5	5	5	5	4	5	5	5	3	5	4	3	3	3	2	2	2	2	2	2	2	2	1	1	2	2	2	2	2	2	2.50	1.80	5.00	4.67	4.33	4.73
201	2022/10/10 11:42:36	4609秒	2	2	2	2	4	5	3	3	2	2	5	2	3	2	3	3	3	3	3	3	2	4	4	3	2	2	1	1	1	3	2	2	2	2	3.10	1.80	3.40	3.00	2.33	3.00
202	2022/10/10 12:58:47	228秒	2	2	4	4	4	4	3	3	2	2	2	3	2	3	4	4	4	4	4	4	4	4	4	4	3	3	3	3	3	3	3	3	3	3	4.00	3.00	3.60	2.00	2.67	2.91
203	2022/10/10 14:17:01	856秒	2	2	4	3	3	3	3	3	3	3	3	3	3	3	4	4	4	4	4	4	4	4	4	4	2	2	2	2	2	2	2	2	2	2	4.00	2.00	3.00	3.00	3.00	3.00
204	2022/10/10 14:38:48	723秒	2	2	4	2	2	2	2	2	2	2	2	2	2	2	4	3	4	4	4	4	4	4	4	4	3	2	3	2	2	3	2	2	3	2	3.90	2.40	2.00	2.00	2.00	2.00
205	2022/10/10 14:39:38	356秒	2	2	2	2	2	2	2	2	2	2	2	1	1	2	5	5	5	5	5	5	5	5	5	5	4	4	4	4	3	4	4	4	4	4	5.00	3.90	2.00	2.00	1.33	1.82
206	2022/10/10 15:39:23	407秒	2	3	3	3	3	3	3	3	3	3	4	3	4	4	2	2	2	2	2	2	2	2	2	2	2	3	1	1	1	1	1	1	1	1	2.00	1.30	3.00	3.33	3.67	3.27
207	2022/10/10 20:07:13	487秒	2	2	2	5	5	5	5	5	5	5	5	5	5	5	1	1	1	1	1	1	1	1	1	1	1	1	1	1	1	1	1	1	1	1	1.00	1.00	5.00	5.00	5.00	5.00
208	2022/10/10 22:34:36	499秒	2	2	3	4	5	5	3	3	3	3	3	5	5	5	3	3	4	3	5	4	4	4	4	3	4	4	2	2	2	4	4	2	3	3	3.70	3.00	4.00	3.00	5.00	4.00
209	2022/10/11 14:16:47	351秒	2	3	2	3	3	3	3	3	3	3	3	4	4	3	3	3	4	3	4	3	3	3	3	3	3	2	2	2	2	2	2	2	2	2	3.20	2.10	3.00	3.00	3.67	3.18
210	2022/10/11 20:00:30	312秒	2	2	4	2	3	3	3	4	2	2	3	2	2	2	4	5	4	4	3	4	3	4	3	4	3	3	3	3	3	3	3	3	3	3	3.80	3.00	3.00	2.33	2.00	2.55
211	2022/10/12 8:16:43	531秒	2	3	2	4	4	4	3	3	3	3	5	3	3	4	3	3	3	3	3	3	3	3	3	3	2	1	1	1	1	1	2	1	1	1	3.00	1.20	3.60	3.67	3.33	3.55
212	2022/10/12 19:07:15	424秒	2	3	3	3	3	3	3	3	3	3	3	2	2	2	4	4	4	4	4	4	4	4	4	4	3	3	3	2	3	3	3	2	3	2	4.00	2.70	3.00	3.00	2.00	2.73
213	2022/10/15 16:47:51	235秒	2	3	1	5	5	5	3	2	3	3	1	2	2	2	5	5	5	3	4	4	3	4	4	4	3	3	3	3	3	3	3	3	3	3	4.10	3.00	4.00	2.33	2.00	3.00
